# Use of the Perceval Sutureless Valve in Patients with a Type 0 Bicuspid Aortic Valve

**DOI:** 10.1016/j.cjco.2025.11.013

**Published:** 2025-11-21

**Authors:** Ryaan EL-Andari, Sabin J. Bozso, Liang Cao, Zhaoyun Cheng, Shaohua Wang

**Affiliations:** a Division of Cardiac Surgery, Department of Surgery, University of Alberta, Edmonton, Alberta, Canada; b Division of Cardiovascular Surgery, Sanger Heart and Vascular Institute, Charlotte, North Carolina, USA; c Division of Cardiac Surgery, 2nd Xiangya Hospital, Changsha, China; d Division of Cardiac Surgery, Fuwai Central China Hospital, Zhengzhou, China

**Keywords:** cardiac surgery, Perceval valve, bicuspid aortic valve

## Abstract

The valve implantation technique for using a Perceval valve in a type 0 bicuspid aortic valve (BAV) is modified with 3 guiding sutures being placed 120˚ apart, creating 3 neo-sinuses. Six patients with a type 0 BAV underwent aortic valve replacement with the Perceval sutureless valve. The mean age was 68.0 years, and 3 patients were male. No perivalvular leak occurred postoperatively, and no in-hospital mortality occurred in this cohort. The Perceval valve can be implanted safely in patients with a type 0 BAV, although careful consideration of patient anatomy intraoperatively is required to determine the required surgical modifications.

## Résumé

La technique d'implantation de la valve Perceval dans une valve aortique bicuspide (VAB) de type 0 a été modifiée en plaçant 3 sutures de guidage espacées de 120°, créant ainsi 3 néo-sinus. Six patients atteints d'une bicuspidie aortique de type 0 ont bénéficié d'un remplacement valvulaire aortique avec une valve Perceval sans suture. L'âge moyen était de 68,0 ans et 3 patients étaient des hommes. Il n'y a eu aucune fuite périvalvulaire postopératoire et aucun décès intrahospitalier pour cette cohorte. La valve Perceval peut être implantée en toute sécurité chez les patients atteints d'une VAB de type 0, bien qu'il soit nécessaire d'examiner attentivement l'anatomie du patient pendant l'opération afin de déterminer les ajustements chirurgicaux requis.

**Figure 1:**
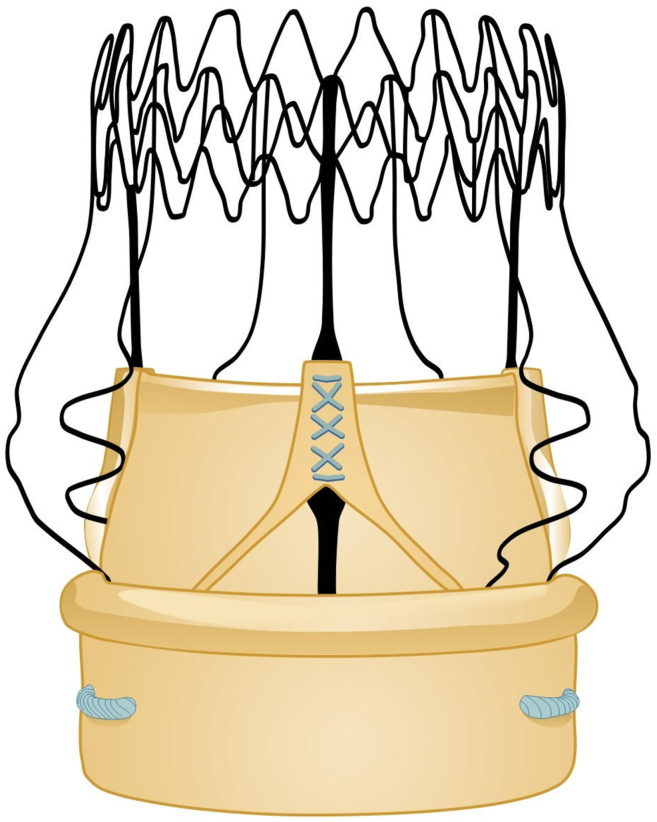
Illustration of the Perceval sutureless valve (Corcym, London).

Sutureless and rapid deployment valves (RDVs) for aortic valve replacement (AVR) have gained popularity in recent years. By reducing operative times, facilitating minimally invasive approaches to AVR, and providing beneficial hemodynamic profiles, RDVs have been applied in a variety of settings.^1^, ^2^, ^3^, ^4^, ^5^ The Perceval valve (Corcym, London; Fig. 1) is among the most common RDVs and has been in use since 2007. The device is comprised of a valve constructed from bovine pericardium and a self-expanding nitinol stent with 2 rings connected by struts.^1^^,^^3^

Although the Perceval valve has been utilized in a variety of settings, its use has been limited in patients with bicuspid aortic valves (BAVs), which is among the most common causes of severe aortic stenosis. Given the asymmetric, elliptical, and often large annulus commonly identified in 2 sinus BAVs, concerns have been raised regarding incomplete sealing, perivalvular leak, and valve migration when RDVs are used in a BAV.^6^, ^7^, ^8^, ^9^, ^10^ Although several series have been described, these have been limited to select reports, as a type 0 BAV largely has been considered a contraindication for use of the Perceval valve, given the aforementioned concerns with its use in this setting. Herein, we describe a cohort of patients with a type 0 BAV undergoing AVR with the Perceval valve.

## Methods

### Ethics

Ethics approval was obtained from the local research ethics board on November 5, 2024, for study ID Pro00147430. The cases included in this report were collected from multiple centres in China from 2022-2024 as part of an online proctoring collaboration.^11^ All data received by the authors from each participating institution were anonymized to protect the identity of the patients, and each centre was responsible for obtaining ethics and/or consent based on their own guidelines.

### Modifications for a BAV

Preoperative assessment on imaging is necessary to ensure that the described technique will be appropriate for the individual patient. Preoperative assessment of valve size on computed tomography scan should ensure that the maximum diameter of the valve does not exceed 27 mm, which is the size of the extra-large size Perceval. The surgeon should also evaluate the available imaging to identify whether an elliptical annulus is present, which may require further modification to the surgical technique or a change of valve type.

The Perceval valve can be implanted through a variety of surgical approaches, including conventional sternotomy, hemisternotomy, and right anterior thoracotomy. In this cohort, all cases were performed via sternotomy. A transverse aortotomy is made 1-2 cm above the sino-tubular junction. and the annulus requires careful debridement to remove any calcium, especially any deposits that protrude into the aortic orifice where the valve will be deployed. Once adequately debrided, 3 guiding sutures are placed at the nadirs. The valve is sized using sizers specific to the Perceval valve that have white and transparent ends. With an appropriately sized valve, the transparent end of the sizer will pass easily through the aortic annulus. and the white end will encounter resistance at the annulus.^5^ Appropriate valve sizing, including avoidance of valve oversizing, is imperative to ensure long-term durability of the valve, as oversizing results in pinwheeling of the valve leaflets, increased gradients across the valve, greater strain on the leaflets, and increased risk of early valve failure. Previous literature has demonstrated low rates of structural valvular deterioration with the Perceval valve, including > 95% freedom from structural valvular deterioration at 10-year follow-up evaluation.^5^^,^^12^^,^^13^

**Figure 2:**
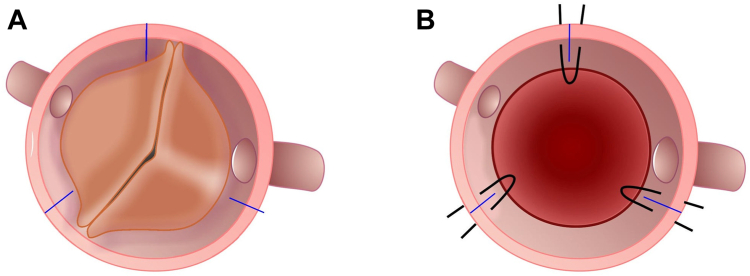
A bicuspid aortic valve with lines marked to represent (**A**) the nadir and 2 other marks 120˚ apart and (**B**) the aortic annulus with the valve excised and guiding sutures in place.

Once the valve has been deployed, these guiding sutures are removed. A balloon is then expanded inside the valve to ensure that it is completely deployed. In a standard AVR with a Perceval valve, 3 guiding sutures are placed at the nadirs, 120˚ apart. In contrast, with the type 0 BAV, the 2 nadirs are ∼180˚ apart, resulting in 2 sinuses. To address this issue, the sutures still must be placed 120˚ apart, creating 3 neo-sinuses. To accomplish this, following excision of the native valve leaflets and debridement of the aortic annulus, the Perceval valve sizer is placed in the aortic annulus with 1 of the 3 markers at 1 of the 2 nadirs, between the 2 coronary ostia (Fig. 2A). Three marks are then made with a marking pen at the 1 nadir, and then 2 more are made 120˚ apart. Once adequate locations have been chosen, the 3 guiding sutures then are placed 2-3 mm below the annulus, creating 3 neo-sinuses, and are passed through the guiding sutures on the Perceval valve (Fig. 2B). The valve is then placed and deployed (Supplemental Fig. S1, A and B). In the case of a very elliptical annulus, previous techniques have been described to restore circularity of the annulus, including placing a plication suture at one of the commissures, which is tied before placement of the valve to remodel the annulus.^6^^,^^14^^,^^15^

Although a satisfactory result after the initial deployment is common, the Perceval valve is easily recaptured and redeployed, and the surgeon should have a low threshold to redeploy the valve if pinwheeling of the leaflets, perivalvular leak, or high gradients are identified post-deployment.

## Results

Six patients with a type 0 BAV underwent AVR with the Perceval valve. The 6 patients included in this study were obtained from 5 hospitals. The number of valve surgeries conducted at each of the 5 hospitals over the study period ranged from 600-10,000, with 4-40 Perceval valves implanted at each. The mean patient age was 68.0 years, and 3 patients (50%) were male (Table 1). The indication for surgery was aortic stenosis in all cases. All patients had a type 0 bicuspid aortic valve. Two patients underwent a concomitant ascending aortic replacement, and the remaining patients underwent isolated AVR. Cardiopulmonary bypass times ranged from 82-115 minutes and cross-clamp times ranged from 39-88 minutes. The valve sizes of the implanted valves were medium (3), large (2), and extra-large (1). No perivalvular leak occurred postoperatively, in all cases, and no in-hospital mortality occurred in this cohort.

**Table 1:** Baseline demographics and operative details of the included study population

Patient Number	Age, y	Sex	Surgery	Valve size	Indication	Cardiopulmonary bypass time, min	Cross-clamp time, min
1	59	F	Isolated AVR	Med	AS	110	69
2	66	F	Isolated AVR	Med	AS	—	—
3	72	M	AVR, ascending aortic replacement	L	AS, aortic aneurysm	115	88
4	69	F	Isolated AVR	Med	AS	82	47
5	72	M	AVR, ascending aortic replacement	XL	AS, aortic aneurysm	82	47
6	70	M	Isolated AVR	L	AS	84	39

Patients all had type 0 bicuspid valve. No patients had postoperative perivalvular leak or pacemaker insertion. The surgical approach used for all patients was full sternotomy.

AS, aortic stenosis; AVR, aortic valve replacement; F, female; L, large; M, male; Med, medium; XL, extra large.

## Discussion

BAV is among the most common causes of early aortic valve failure and required AVR. Anatomic challenges to sutureless or transcatheter valve implantation exist with type 0 BAV, including an asymmetrical and often large annulus. At the time of valve replacement, intraoperative modifications to technique or anatomic modifications can help facilitate the safe and effective use of sutureless valves. These modifications include changing suture placement, identifying an appropriate initial placement of a valve suture between the 2 coronary sinuses, and utilizing the Perceval valve sizer to ensure the subsequent sutures are 120° apart while avoiding coronary obstruction. Despite these possibilities, utilization of the Perceval valve in patients with a type 0 BAV has been limited, with only small numbers of patients reported in select studies in the literature to date.^6^^,^^14^^,^^15^

Similar to the use of transcatheter valves in BAV, asymmetric commissures and an elliptical annulus pose significant challenges for the implantation of RDVs. In contrast to transcatheter aortic valve replacement, surgical replacement with the Perceval valve allows for implantation in patients with a broader range of anatomy, as the implantation can be modified intraoperatively to suit individual patient anatomy, as with the placement of the guiding sutures under direct vision for the Perceval.^6.15^ However, patients with extreme anatomy, such as very elliptical or large annuli, may not be appropriate for this technique and may instead require a sutured valve or aortic root intervention. With respect to preoperative evaluations of valve size, computed tomography is not required, as the valve is measured intraoperatively, the technique can be modified to suit the patient's anatomy, and in cases in which a Perceval valve is deemed not appropriate, a different valve can be selected at the time of the surgery. In this cohort, all patients underwent successful AVR with the Perceval valve, with no reports of postoperative perivalvular leak. This result further confirms the safety of the use of the Perceval valve in patients with type 0 BAVs, with specific consideration of patient anatomy intraoperatively.

### Limitations

The sample size included in this report is small, and further reports of sutureless valve use in this patient population are warranted. Additionally, as the Perceval valve has not been used in patients with type 0 BAVs previously, long-term data are lacking and patients in this population who undergo this procedure require long-term follow-up to verify valve durability.

## Conclusion

The Perceval valve can be implanted safely in patients with a type 0 BAV, although careful consideration of patient anatomy intraoperatively is required to determine the required modifications to the surgical technique.
