# Supplementary material for: Use of the Perceval Sutureless Valve in Patients with a Type 0 Bicuspid Aortic Valve
Source: CJC Open. 2025 Nov 21;8(3):332–5. doi: 10.1016/j.cjco.2025.11.013 (PMC12983244; doi:10.1016/j.cjco.2025.11.013)

Supplemental Figures

Supplemental Figure S1. Perceval valve during valve deployment and following implantation.

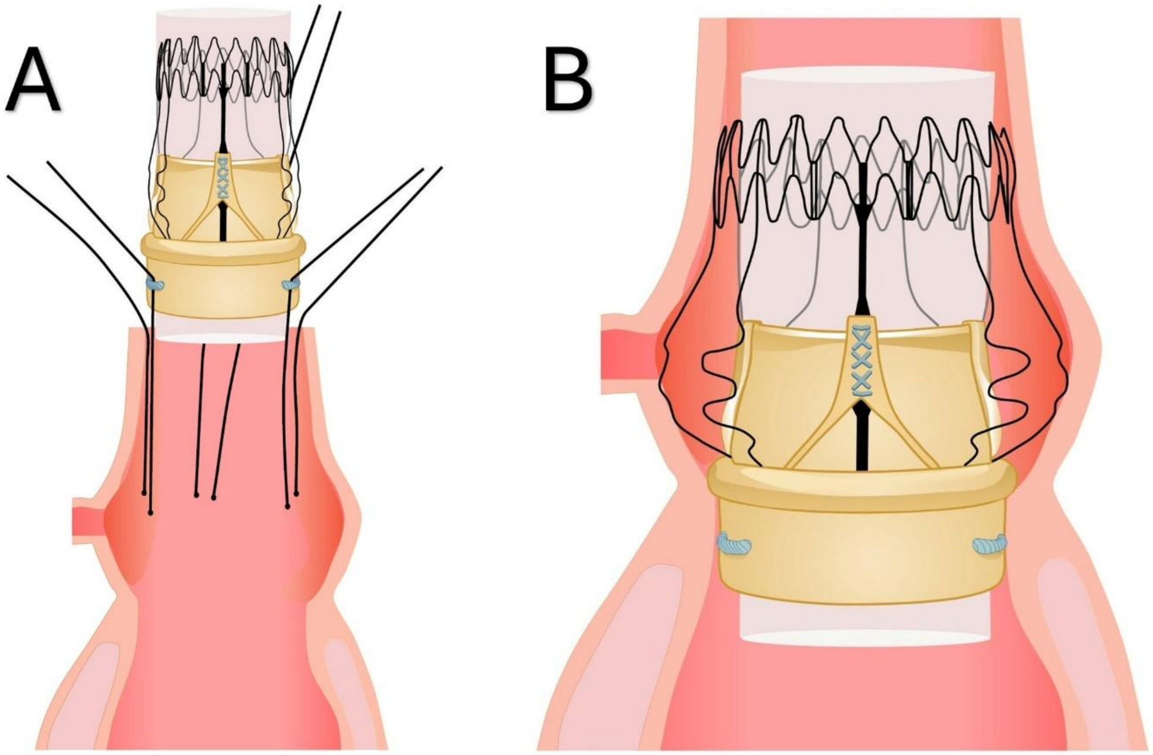

Supplement: Supplementary Material [file mmc1.pdf]
